# Supplementary material for: Does gestational diabetes increase the risk of maternal kidney disease? A Swedish national cohort study
Source: PLoS One. 2022 Mar 10;17(3):e0264992. doi: 10.1371/journal.pone.0264992 (PMC8912264; doi:10.1371/journal.pone.0264992)
Supplement: S1 Table — (DOCX) [file pone.0264992.s001.docx]

**Supplemental Table S1. ICD codes used for disease definitions**

|  | **ICD-9 codes**  **(1987-1996)** | **ICD-10 codes**  **(1997-2013)** |
| --- | --- | --- |
| Any pre-existing chronic/end-stage kidney disease, congenital or genetic causes of renal disease (for exclusion) | 403-404, 580-589, 753, V42A, V45B, V56A, V56W | N00-N08, N10-N19, P960, Q271, Q272, Q60-Q63, Q878, Z49, Z992, Z940, T861 |
| Chronic kidney disease (CKD)  (Outcome) | 250D, 403-404, 581-583, 585-588, V42A, V45B, V56A, V56W | E102, E112, I12-I13, I150-I151, N01-N06, N08, N11-N13, N15-16, N18-19, Z49, Z992, Z940, T861 |
| Tubulointerstitital CKD | - | N11-N12, N15-N16 |
| Glomerular/proteinuric CKD | 581-583 | N01-N06, N08 |
| Hypertensive CKD | 403-404 | I12-I13, I150-I151 |
| Diabetic CKD | 250D | E102, E112 |
| Other/unspecified CKD | 585-588, V42A, V45B, V56A, V56W | N13, N18-N19, Z49, Z992, Z940, T861 |
| End-stage kidney disease (ESKD) (Outcome) | V42A, V45B, V56A, V56W | N185, Z49, Z992, Z940, T861 |
| Cardiovascular disease | 393-398  410-436 | I16-I64  G45 |
| Essential hypertension | 401-405 | I10-I15 |
| Diabetes (type 1 or type 2) | 250 | E10-E14 |
| Systemic lupus erythematosus | 710A | M32 |
| Systemic sclerosis | 710B | M34 |
| Vasculitis | 446 | M31 |
| Haemoglobinopathies | 282-283 | D56-D59 |
| Coagulopathies | None | D68 |
| Preeclampsia | 642E, 642F, 642G | O140, O141, O141A,  O141B, O141C, O141X, O142, O149, O150, O151, O152, O159 |
| Gestational diabetes | 648W | O244 |
