# Supplementary material for: Does gestational diabetes increase the risk of maternal kidney disease? A Swedish national cohort study
Source: PLoS One. 2022 Mar 10;17(3):e0264992. doi: 10.1371/journal.pone.0264992 (PMC8912264; doi:10.1371/journal.pone.0264992)
Supplement: S2 Table — (DOCX) [file pone.0264992.s002.docx]

**Supplemental Table S2. Demographic changes over time among women whose first delivery occurred in Sweden between 1987 and 2012**

|  | **Births in Sweden** | | | | |
| --- | --- | --- | --- | --- | --- |
|  | **1987-1991** | **1992-1996** | **1997-2001** | **2002-2006** | **2007-2012** |
| Number of singleton births* | 533,078 | 499,856 | 399,119 | 442,219 | 584,308 |
| Incidence rate of GDM (per 100,000 births) | 562 | 646 | 803 | 917 | 1,116 |
| Mean (± sd) age at delivery | 26**·**4 ± 4**·**7 | 27**·**8 ± 4**·**7 | 29**·**1 ± 4**·**8 | 30**·**0 ± 5**·**0 | 30**·**2 ± 5**·**3 |
| % mothers affected by antenatal obesity** | 3**·**9 | 6**·**4 | 9**·**0 | 10**·**7 | 12**·**1 |
| % mothers born outside of Sweden | 14**·**4 | 17**·**3 | 13**·**2 | 11**·**2 | 13**·**5 |

GDM, gestational diabetes

*after exclusions applied, i.e. only women whose first birth happened during or after 1987 and excluding multiple pregnancy, pre-pregnancy medical comorbidities (renal disease, cardiovascular disease, chronic hypertension, systemic lupus erythematosus, systemic sclerosis, coagulopathies, vasculitides, haemoglobinopathies, type 1 or 2 diabetes mellitus at baseline)

**after missing data addressed using multiple imputation
