# Supplementary material for: Does gestational diabetes increase the risk of maternal kidney disease? A Swedish national cohort study
Source: PLoS One. 2022 Mar 10;17(3):e0264992. doi: 10.1371/journal.pone.0264992 (PMC8912264; doi:10.1371/journal.pone.0264992)
Supplement: S3 Table — (DOCX) [file pone.0264992.s003.docx]

**Supplemental Table S3.** **Time to diagnosis of chronic kidney disease (CKD) subtypes among women whose first live birth occurred between 1987 and 2012 in Sweden, stratified by exposure to GDM**

|  | | **n** | **Time to diagnosis (years)** | |
| --- | --- | --- | --- | --- |
|  | |  | **Median (IQR)** | **Log-rank, p** |
| **Overall CKD** | |  |  |  |
| No GDM | | 5,725 | 6.7 (2.8-11.4) | <0.001 |
| Exposed to GDM | | 154 | 5.9 (2.6-12.0) |  |
| **1.** | **Tubulointerstitial CKD** |  |  |  |
|  | No GDM | 1,325 | 6.0 (2.8-11.3) | 0.983 |
|  | Exposed to GDM | 18 | 7.0 (3.2-10.2) |  |
| **2.** | **Glomerular/proteinuric CKD** |  |  |  |
|  | No GDM | 1,755 | 5.0 (2.2-10.3) | 0.32 |
|  | Exposed to GDM | 45 | 4.9 (2.0-9.3) |  |
| **3.** | **Hypertensive CKD** |  |  |  |
|  | No GDM | 132 | 9.8 (4.6-15.9) | 0.29 |
|  | Exposed to GDM | 6 | 13.9 (6.7-17.0) |  |
| **4.** | **Diabetic CKD** |  |  |  |
|  | No GDM | 88 | 7.0 (2.7-12.6) | 0.66 |
|  | Exposed to GDM | 49 | 5.7 (3.0-9.6) |  |
| **5.** | **Other/unspecified CKD** |  |  |  |
|  | No GDM | 2,425 | 9.9 (2.8-15.1) | 0.137 |
|  | Exposed to GDM | 36 | 6.7 (2.8-13.3) |  |
