# Supplementary material for: Does gestational diabetes increase the risk of maternal kidney disease? A Swedish national cohort study
Source: PLoS One. 2022 Mar 10;17(3):e0264992. doi: 10.1371/journal.pone.0264992 (PMC8912264; doi:10.1371/journal.pone.0264992)
Supplement: S4 Table — (DOCX) [file pone.0264992.s004.docx]

**Supplemental Table S4. Hazard ratios for maternal kidney disease by history of gestational diabetes and delivery of a large for gestational age infant, among women whose first birth occurred between 1987 and 2012 in Sweden**

|  | **Chronic kidney disease (N=5,879)** | | |
| --- | --- | --- | --- |
|  | **n** | **Age-adjusted** | **Fully adjusted** |
|  |  | **HR (95% CI)** | **HR (95% CI)** |
| None | 5,428 | 1**·**0 | 1**·**0 |
| GDM only | 105 | 2**·**00 (1**·**65-2**·**42) | 1**·**58 (1**·**31-1**·**93) |
| LGA only | 297 | 1**·**60 (1**·**44-1**·**78) | 1**·**43 (1**·**29-1**·**60) |
| GDM and LGA | 49 | 4**·**72 (3**·**56-6**·**26) | 3**·**03 (2**·**28-4**·**03) |
|  | **End-stage kidney disease (N=228)** | | |
| None | 203 | 1**·**0 | 1**·**0 |
| GDM only | 12 | 5**·**61 (3**·**13-10**·**07) | 3**·**78 (2**·**08-6**·**87) |
| LGA only | 7 | 1**·**42 (0**·**81-2**·**49) | 1**·**37 (0**·**78-2**·**42) |
| GDM and LGA | 6 | 14**·**64 (6**·**49-33**·**02) | 8**·**37 (3**·**64-19**·**23) |

GDM, gestational diabetes; LGA, large for gestational age

Hazard ratios represent separate Cox regression models for associations between gestational diabetes and maternal chronic kidney disease or end-stage kidney disease. In all models, gestational diabetes and/or subsequent type 2 diabetes was a time-dependent variable, where maternal exposure status was based on the date of first affected delivery.

Fully adjusted models were adjusted for maternal age, country of origin, maternal education, parity, antenatal body mass index (BMI), smoking, gestational weight gain and maternal exposure to preeclampsia (time-dependent covariate), stratified by year of delivery. Women with pre-pregnancy history of renal disease, cardiovascular disease, diabetes, hypertension, systemic lupus erythematosus, coagulopathies, haemoglobinopathies and vasculitis were excluded at baseline.
