# Supplementary material for: Does gestational diabetes increase the risk of maternal kidney disease? A Swedish national cohort study
Source: PLoS One. 2022 Mar 10;17(3):e0264992. doi: 10.1371/journal.pone.0264992 (PMC8912264; doi:10.1371/journal.pone.0264992)
Supplement: S5 Table — (DOCX) [file pone.0264992.s005.docx]

**Supplemental Table S5. Effect modification by ethnicity and antenatal obesity status of the association between gestational diabetes and/or type 2 diabetes and maternal renal disease in women whose first birth occurred between 1987 and 2012 in Sweden**

|  | **Chronic kidney disease (N=5,879)** | | | **End-stage kidney disease (N=228)** | | |
| --- | --- | --- | --- | --- | --- | --- |
|  | **Age-adjusted** | **Fully adjusted** | **p interaction** | **Age-adjusted** | **Fully adjusted** | **p interaction** |
|  | **HR (95% CI)** | **HR (95% CI)** |  | **HR (95% CI)** | **HR (95% CI)** |  |
| **Born in Sweden** |  |  |  |  |  |  |
| None | 1**·**0 | 1**·**0 | 0**·**004 | 1**·**0 | 1**·**0 | 0**·**119 |
| GDM only | 1**·**45 (1**·**12-1**·**86) | 1**·**11 (0**·**86-1**·**44) |  | 2**·**42 (0**·**77-7**·**60) | 1**·**33 (0**·**58-4**·**19) |  |
| T2DM only | 23**·**15 (20**·**77-25**·**80) | 19**·**84 (17**·**76-22**·**15) |  | 93**·**92 (66**·**75-132**·**15) | 69**·**90 (48**·**97-99**·**78) |  |
| GDM + T2DM | 26**·**82 (20**·**04-35**·**88) | 14**·**67 (10**·**91-19**·**73) |  | 115**·**01 (50**·**50-261**·**92) | 82**·**92 (35**·**67-192**·**74) |  |
| **Born outside of Sweden** |  |  |  |  |  |  |
| None | 1**·**0 | 1**·**0 |  | 1**·**0 | 1**·**0 |  |
| GDM only | 1**·**22 (0**·**78-1**·**90) | 1**·**06 (0**·**68-1**·**67) |  | 3**·**19 (0**·**98-10**·**35) | 2**·**43 (0**·**73-8**·**06) |  |
| T2DM only | 33**·**16 (26**·**08-42**·**15) | 27**·**78 (21**·**76-35**·**48) |  | 36**·**65 (14**·**29-94**·**02) | 26**·**77 (10**·**21-70**·**23) |  |
| GDM + T2DM | 56**·**59 (38**·**46-83**·**26) | 44**·**32 (29**·**87-65**·**75) |  | 226**·**96 (93**·**91-548**·**51) | 186**·**71 (72**·**48-463**·**41) |  |
|  |  | | |  | | |
| **Obese** |  |  |  |  |  |  |
| None | 1**·**0 | 1**·**0 | 0**·**056 | 1**·**0 | 1**·**0 | 0**·**181 |
| GDM only | 0**·**95 (0**·**61-1**·**48) | 0**·**78 (0**·**50-1**·**22) |  | 5**·**71 (1**·**93-16**·**92) | 5**·**27 (1**·**75-15**·**87) |  |
| T2DM only | 23**·**57 (18**·**48-30**·**06) | 19**·**60 (15**·**31-25**·**09) |  | 56**·**52 (20**·**50-155**·**81) | 43.68 (15**·**27-124**·**98) |  |
| GDM + T2DM | 46**·**06 (32**·**26-65**·**76) | 26**·**47 (18**·**29-38**·**29) |  | 168**·**47 (60**·**85-466**·**42) | 172**·**43 (57**·**85-513**·**99) |  |
| **Non-obese** |  |  |  |  |  |  |
| None | 1**·**0 | 1**·**0 |  | 1**·**0 | 1**·**0 |  |
| GDM only | 1**·**48 (1**·**15-1**·**91) | 1**·**27 (0**·**99-1**·**64) |  | 1**·**35 (0**·**33-5**·**46) | 1**·**01 (0**·**25-4**·**08) |  |
| T2DM only | 23**·**76 (21**·**32-26**·**49) | 20**·**82 (18**·**65-23**·**24) |  | 82**·**19 (58**·**83-114**·**82) | 62**·**27 (44**·**06-87**·**99) |  |
| GDM + T2DM | 24**·**66 (18**·**05-33**·**68) | 18**·**25 (13**·**35-24**·**95) |  | 124**·**60 (58**·**14-267**·**03) | 89**·**81 (41**·**44-194**·**67) |  |

Hazard ratios represent separate Cox regression models for associations between gestational diabetes and maternal chronic kidney disease or end-stage kidney disease. In all models, gestational diabetes and/or subsequent type 2 diabetes was a time-dependent variable, where maternal exposure status was based on the date of first affected delivery.

Fully adjusted model adjusted for maternal age, country of origin, maternal education, parity, antenatal body mass index (BMI), smoking, gestational weight gain and maternal exposure to preeclampsia (time-dependent covariate), stratified by year of delivery. Women with pre-pregnancy history of renal disease, cardiovascular disease, diabetes, hypertension, systemic lupus erythematosus, coagulopathies, haemoglobinopathies and vasculitis were excluded at baseline.
