# Supplementary material for: Does gestational diabetes increase the risk of maternal kidney disease? A Swedish national cohort study
Source: PLoS One. 2022 Mar 10;17(3):e0264992. doi: 10.1371/journal.pone.0264992 (PMC8912264; doi:10.1371/journal.pone.0264992)
Supplement: S1 Fig — (DOCX) [file pone.0264992.s006.docx]

**N=2,703,072**

Total pregnancies recorded in Sweden from 1 January 1987 to 31 December 2012

**N=244,492**

Pregnancies excluded from dataset for the following reasons:

| Pre-pregnancy diseases | |  |
| --- | --- | --- |
|  | *Renal disease* | n=13,241 |
|  | *Diabetes* | n=11,821 |
|  | *Hypertension* | n=9,470 |
|  | *Cardiovascular disease* | n=2,343 |
|  | *Systemic lupus erythematosus* | n=2,120 |
|  | *Systemic sclerosis* | n=30 |
|  | *Coagulopathy* | n=4,238 |
|  | *Hemoglobinopathy* | n=1,381 |
|  | *Vasculitides* | n=51 |
| Multiple pregnancy | | n=105,200 |
| Implausible or incomplete information on date of delivery | | n=123 |
| Implausible birth weight for gestational age | | n=8,389 |
| Died or emigrated before date of first delivery recorded | | n=86,085 |

**N=2,458,580**

Eligible pregnancies in Sweden from 1 January 1987 to 31 December 2012

**Supplemental Figure S1. Flow chart illustrating construction of study cohort**
